# Nature-based mindfulness programs using virtual reality to reduce pediatric perioperative anxiety: a narrative review

**DOI:** 10.3389/fped.2024.1334221

**Published:** 2024-01-12

**Authors:** Brandon Benchimol-Elkaim, Bassam Khoury, Argerie Tsimicalis

**Affiliations:** ^1^ Department of Educational and Counselling Psychology, McGill University, Montreal, QC, Canada; ^2^ Ingram School of Nursing, McGill University, Montreal, QC, Canada; ^3^ Shriners Hospital for Children, Montreal, QC, Canada

**Keywords:** virtual reality, nature, mindfulness-based intervention (MBI), perioperative anxiety, pediatric

## Abstract

Over 75% of pediatric surgery patients experience preoperative anxiety, which can lead to complicated recoveries. Current interventions are less effective for children over 12 years old. New interventions, like mindfulness-based ones (MBIs), are needed to address this issue. MBIs work well for reducing mental health symptoms in youth, but they can be challenging for beginners. Virtual reality (VR) nature settings can help bridge this gap, providing an engaging 3-D practice environment that minimizes distractions and enhances presence. However, no study has investigated the combined effects of mindfulness training in natural VR settings for pediatric surgery patients, creating a significant gap for a novel intervention. This paper aims to fill that gap by presenting a narrative review exploring the potential of a nature-based mindfulness program using VR to reduce pediatric preoperative anxiety. It begins by addressing the risks of anxiety in children undergoing surgery, emphasizing its impact on physical recovery, and supporting the use of VR for anxiety reduction in hospitals. The review then delves into VR's role in nature and mindfulness, discussing theoretical concepts, clinical applications, and effectiveness. It also examines how the combination of mindfulness, nature, and VR can create an effective intervention, supported by relevant literature. Finally, it synthesizes the existing literature's limitations, findings, gaps, and contradictions, concluding with research and clinical implications.

## Introduction

The perioperative period begins at the moment from which a patient enters the hospital for surgery and lasts until they are released to go home (1). This time period can result in elevated anxiety levels, which is especially true in children (2). Typically, children will experience peak anxiety levels in the preoperative period (the time period from entering the hospital to the administration of anesthesia), as they await the administration of anesthesia before surgery (3). This peak in anxiety represents a major problem, as more than 75% of pediatric surgery patients experience preoperative anxiety (anxiety regarding the impending surgical experience) (4). This anxiety manifests itself as feelings of tension, nervousness, and fear during their hospital stay (5). These feelings are extremely traumatic for patients, resulting in around 16% of children developing post-traumatic stress disorder following surgery (6). Even more concerning, preoperative anxiety can also have severe consequences on physical health. Several studies report that high levels of preoperative anxiety leads to a more painful, slower, and complicated recovery (7, 8).

A variety of interventions have been developed over the years to help reduce preoperative anxiety. Among these interventions, interventions targeting preoperative anxiety can have significant positive effects on the patient (5, 9, 10). The interventions are diverse, ranging from educational preparatory puppet shows to child animators (e.g., clowns, magicians, mascots, etc.) being present on hospital floors. While effective, these interventions are not without their shortfalls. Most of these interventions can only be done prior to the administration of anesthesia, due to restricted access in the operating room, leaving the children without interventions available during the peak of their anxiety. These interventions also significantly lose their effectiveness with children over the age of 12 (5). These interventions are also resource-intensive programs requiring full-time staff in increasingly human resource strapped systems. Additionally, these interventions are dependent on an adult taking the lead in helping a child reduce their anxiety. Thus, these interventions fail to prepare children with skills to self-regulate their anxiety for their long surgical journey. As such, there is a growing need for new self-regulated techniques to reduce preoperative anxiety in children.

One novel way to reduce preoperative anxiety in children in a self-regulated matter could be by offering mindfulness-based interventions (MBIs). Mindfulness is the process of purposefully, and with acceptance, paying attention non-judgmentally to the present moment (11). MBIs are behavioural interventions that teach mindfulness, following a mind-body medicine view of health by integrating physical, cognitive-affective, behavioural, and social elements (11). Typically, MBIs consist of meditation and accompanied by a variety of mindfulness techniques such as guided imagery and breathing exercises. Data strongly supports the use of MBIs, and further data has shown that they are effective at reducing mental health symptoms in youth and can provide children with long-lasting skills that can be used independently (12). Despite the potential benefits of MBIs, many children do not practice mindfulness. Cultivating mindfulness can be challenging and demanding for novices, and newcomers may experience boredom and wandering thoughts due to the lack of sensory input involved.

Virtual reality (VR) has gained attention in recent years as a potential tool for mindfulness training. VR is a simulation tool that enables users to be fully immersed in a 3-D virtual environment, allowing for a more engaging and interactive experience (13). VR can offer an interesting and immersive setting to practice mindfulness by limiting distractions from the real world and increasing a sense of presence (14). By removing external distractions, users can focus more intently on the present moment and their internal experiences, which is a fundamental aspect of mindfulness. Moreover, VR has the potential to induce specific emotions at varying levels of arousal and valence, which could be useful for mindfulness practice (13). For instance, VR environments could be designed to evoke positive emotions such as joy, gratitude, or contentment, which are associated with increased well-being and decreased stress levels (15).

To effectively utilize VR technology for mindfulness training, it is essential to design the virtual environments carefully. Incorporating natural settings such as forests, mountains, or beaches into VR experiences could enhance the therapeutic efficacy of mindfulness training. Exposure to natural environments can improve mood and decrease stress levels, so incorporating these settings into VR could enhance the benefits of mindfulness training (16–19).

Despite the immense potential, no study to date has investigated the combined effects of mindfulness training in natural settings using VR with pediatric patients undergoing surgery, leaving a large gap for a novel intervention. To address this gap, this paper presents the findings of a narrative review that sought to explore the potential for a nature-based mindfulness program using virtual reality in reducing pediatric preoperative anxiety. The review will first focus on the risks associated with anxiety in children undergoing surgery, with a particular emphasis on the impact on physical recovery. This section will also support the validity of using VR as an intervention for reducing anxiety in children and provide a brief overview of the kinds of VR interventions currently being used in hospitals. Subsequently, the review will focus on the use of VR in relation to nature and mindfulness, respectively. Key theoretical conceptualizations, current clinical applications, and therapeutic efficacy will be discussed. The review will then examine the connection between mindfulness and nature, and how the combination of these two elements with VR can lead to an effective intervention. The literature supporting this connection will be brought together in this section. Finally, a synthesis of the limitations, findings, gaps, and contradictions in the existing literature will be discussed and will conclude with research and clinical implications.

## Pediatric preoperative anxiety

Anxiety is an emotion typically associated with feelings of worry, tension, racing thoughts, and physical changes (such as increased heart rate) (20). It contains an affective, behavioural, cognitive, and physiological component. While separate constructs from anxiety, worry and fear often accompany feelings of anxiety. Worry is a future-oriented construct characterized by anticipating a future threat, whereas fear is a present-oriented response to an identified threat (20). Preoperative anxiety is defined as the anxiety experienced in anticipation of surgery (3). This anxiety manifests itself as feelings of tension, nervousness, and fear during their hospital stay (5). This can include a wide range of worries, such as negative and distressing thoughts in anticipation of surgery and fears, such as fearing needles (20). Due to the distressing nature of anxiety, and the worry and fear that comes with it, many will experience a natural response to want to flee or avoid situations, such as avoiding going to the hospital. More physiological manifestations of preoperative anxiety include becoming restless, laboured breathing, trembling, crying, and ceasing communication or play (3). Additionally, some children may unexpectedly urinate, demonstrate heightened muscle tension, or try to evade medical staff. It has been noted that up to 25% of children may require physical restraint during anesthesia induction, a circumstance that can intensify stress levels for both the children and medical personnel involved (3).

Several explanations exist for the root cause of pediatric preoperative anxiety. Most frequently, anxiety stems from the lack of control one has during the perioperative period, along with the unknowns of surgery (4). Children are also substantially worried about the use of needles and induction of anesthesia (4, 21). Additionally, children fear being separated from their parents (22). They also fear the pain and after-effects of surgery.

There are several predictors associated with preoperative anxiety in children. Shy children, those with high IQ, and individuals with poor adaptive abilities are more likely to experience preoperative anxiety (4). Additionally, children with pre-existing anxiety or depression, somatization tendencies, and a fearful temperament may also be at a higher risk. It is worth noting that parental anxiety can also predict preoperative anxiety in children (4). However, there is conflicting evidence regarding the associations between gender, age, and previous medical experiences with preoperative anxiety (4). Some studies suggest that gender does not significantly impact preoperative anxiety levels or the emergence of negative behaviours after surgery. Conversely, other studies suggest that either males or females may be at a higher risk for preoperative anxiety (4, 7, 22–28). Similarly, findings regarding the relationship between age and preoperative anxiety are inconclusive. While certain studies indicate that younger children experience more frequent and prolonged postoperative behavioural problems, other studies contradict these findings and show no significant differences. Notably, regardless of cognitive ability, distress during the anesthesia induction process has remained consistent across different age groups, indicating that anxiety can arise during this stage (4, 23–27, 29–32). Additionally, the impact of negative memories from past hospital experiences, pediatrician visits, or dental visits on preoperative anxiety varies among studies, with some indicating that such memories persist into adolescence and increase pre-operative anxiety. In contrast, others report contradictory results or find no discernible relationship (4, 22, 26, 27, 30, 31, 33, 34).

## Current contexts: addressing a pressing need

When a child experiences preoperative anxiety, it can give rise to a range of complications that have significant implications for their overall well-being and postoperative recovery. These effects frequently last for a significant period of time, with 54% of children showing effects of their preoperative anxiety two weeks later, and 20% still showing signs six months later (4). Firstly, heightened anxiety levels can lead to increased pain experienced during the postoperative period (2, 4, 20, 35–38). Moreover, anxiety is associated with a higher risk of delirium, which can further exacerbate the challenges faced by the child during recovery (4, 20, 35, 37). Additionally, preoperative anxiety may contribute to developing negative behaviours in the postoperative phase, potentially impacting the child's adjustment and overall recovery process (e.g., disobeying medical staff and guidance) (2, 4, 20, 21, 35).

Furthermore, the presence of anxiety can lead to the adoption of maladaptive coping strategies, such as avoidance and repression, which can hinder the child's ability to manage stress and emotional difficulties effectively (38). Anxiety can also disrupt sleep patterns, leading to impaired sleep quality and affecting the child's overall recovery and healing processes (2, 4, 20, 38). It can also disrupt normal eating habits (2, 4, 20).

In terms of medical considerations, preoperative anxiety has been linked to an increased need for pain medication, which can have implications for pain management strategies (2, 4, 20). It is also well-documented that preoperative anxiety can diminish the effects of anesthesia (20). Moreover, the presence of anxiety has been associated with worse surgical outcomes, including delayed wound healing and an increased risk of infection (2, 4, 20). This is due to the fact that anxiety activates the human stress response (HPA axis), which causes an increase in cortisol and epinephrine levels, acting as “natural killer cells” (4). These complications can prolong the recovery period and potentially necessitate additional medical interventions.

Beyond physical consequences, preoperative anxiety can have developmental impacts on the child (2, 20). It can exacerbate separation anxiety, which can be distressing for both the child and their caregivers (2, 4, 20, 37, 38). Furthermore, there is a potential risk for the development of post-traumatic stress disorder (PTSD) (4, 39). Anxiety can also contribute to a slower recovery process overall and has been linked to the occurrence of enuresis (bedwetting) in some cases (4, 20). Preoperative anxiety can also contribute to the development of depression in some children (4). The emotional and psychological toll of anxiety can significantly affect the child's overall well-being and quality of life during and after the surgical experience.

Given these potential complications, addressing and managing preoperative anxiety in children is crucial. Providing appropriate support and interventions to mitigate anxiety can help alleviate the physical, emotional, and developmental burdens associated with the surgical journey, ultimately promoting a smoother and more positive recovery process.

## Virtual reality

Novel approaches are needed to help reduce pediatric preoperative anxiety. One approach available for use is virtual reality. Virtual reality (VR) is an immersive technology that transports users into a computer-generated, three-dimensional world, offering an escape from their current environment (40, 41). This technology has gained recognition among nurses and other health care professionals in child healthcare settings as a valuable tool for distracting children and helping them cope with the pain and anxiety associated with medical procedures (40, 41). A typical VR system consists of a head-mounted display, earphones, a motion tracking system, and hand-held devices for interacting with the virtual world. When wearing the head-mounted display, users can visually explore the virtual environment, while three-dimensional audio and motion tracking provides a real-time update of the virtual world.

## Virtual reality to reduce pediatric preoperative anxiety

The use of virtual reality (VR) to reduce pediatric preoperative anxiety is a relatively new area of research. As a result, the available literature on this topic is limited but growing. A search yielded a total of 8 studies that met the specified criteria, conducted between 2017 and 2021 (42–49). Out of these studies, 7 were randomized controlled trials (RCTs), while one study utilized a pre-post design. Out of the identified studies, six of them employed virtual reality to provide a preview of the operating room to pediatric patients. This approach aimed to familiarize children with the environment and procedures they would encounter during surgery, potentially reducing their anxiety and fear. Additionally, two studies utilized virtual reality games as an intervention method. The age range of participants across these studies spanned from 4 to 12 years old. Encouragingly, 7 of the studies reported a significant decrease in preoperative anxiety following the implementation of virtual reality interventions, while only one found no significant difference between the control group (50). These findings demonstrate the promising potential of VR as an effective tool for alleviating preoperative anxiety in pediatric patients. Despite this potential, none of these interventions provide children with self-regulated techniques to reduce preoperative anxiety. Thus, a major gap still remains in the literature, and there is room in the literature for the investigation of more diverse virtual reality interventions.

While this is an emerging topic with sparse literature, a meta-analysis was recently published evaluating the effectiveness of virtual reality in managing pediatric preoperative anxiety (51). The authors conclude that virtual reality presents a viable, non-pharmacological strategy for addressing anxiety in children before surgery, providing an alternative to analgesic medications and their potential adverse effects. While promising, they also note several issues with the current literature. First, the authors noted a variety of issues with the quality of the literature, and as such, they assigned low-quality ratings to the included studies. Second, they noted some methodological flaws. They point out that future research could benefit by stratifying participants by age group and surgery type to understand the impacts better. They also note a lack of detail regarding virtual reality software used and procedures in the literature (51).

While not the scope of this review, it is important to highlight the prevalence of virtual reality in children's hospitals. This is a technology that is on the rise and is becoming widely embraced by hospital staff. This helps to demonstrate the feasibility of using this technology in hospitals. In a recent review, Addab and colleagues (40) thoroughly examine virtual reality as a distraction intervention to alleviate anxiety and pain in various medical procedures. The authors conducted a comprehensive systematic review of relevant studies published up to 2021. A total of 77 studies were included in the review, covering a range of medical procedures and patient populations. The findings of the review indicate that VR is an effective intervention for reducing anxiety and pain during medical procedures across different patient groups, including children, adults, and individuals with diverse health conditions. VR distraction interventions significantly decreased anxiety and pain levels during procedures such as venipuncture, wound care, dental procedures, and chemotherapy administration. The immersive nature of VR and its ability to divert attention were identified as key factors contributing to its effectiveness (40).

Furthermore, the review highlights the positive impact of VR on patients' overall experience and satisfaction, with many participants reporting reduced fear, increased relaxation, and improved coping during procedures. Feasibility considerations were also addressed, with the majority of studies reporting high acceptability among children, parents and hospital staff. It was also noted that using VR helped improve the facilitation of health practitioners’ tasks. Children would be less tense and fidgety during procedures and less resistant to proposed procedures (40, 52, 53).

## Mindfulness

Mindfulness is an intentional practice and state of heightened consciousness that entails directing one's attention to the current moment, free from judgments or attachments to thoughts, emotions, or sensations that emerge (11). It is frequently characterized as the capacity to bring one's focus to the immediate experience with an open-minded, embracing, and non-reactive mindset. While mindfulness traces its origins to Buddhist customs, it has been modified and incorporated into diverse non-religious contexts and therapeutic methodologies (54).

Mindfulness involves several mechanisms that work together to promote mental well-being. These mechanisms include attention regulation, body awareness, emotion regulation, change in perspective of the self, non-judgement and acceptance, exposure, extinction and reconsolidation, and improved metacognition. In the context of anxiety, emotional regulation, a vital aspect of mindfulness, plays an instrumental role in managing and reacting to distressing emotional experiences (55). Traditional cognitive approaches typically emphasize altering the content of distressing thoughts. However, mindfulness propounds a different strategy, concentrating not on altering or avoiding anxiety-provoking experiences, but on observing and accepting them (55). Through habitual mindfulness practice, individuals suffering from anxiety learn to recognize their feelings as transient states that do not define their identity, offering a perspective shift that facilitates a more measured response to anxiety triggers rather than impulsive reactions. Cultivating a non-judgmental attitude towards one's feelings, mindfulness helps to alleviate the effects of negative emotions and decreases the overall degree of emotional reactivity. Consequently, emotional regulation through mindfulness practice promotes a profound awareness and comprehension of an individual's emotional state, encouraging a healthier engagement with emotions, and contributing to the enhancement of mental health (55). Thus, mindfulness serves as a promising approach in managing anxiety and improving overall well-being.

Mindfulness-based interventions (MBIs) refer to an array of behavioural approaches that seek to foster mindfulness abilities in individuals (56). These interventions acknowledge the interrelatedness of various well-being aspects, which encompass physical, cognitive-affective, behavioural, and social facets. In this regard, MBIs present a holistic framework for enhancing overall wellness via the integration of these dimensions (54, 57–59). By adopting a comprehensive approach to well-being, MBIs offer a promising avenue for promoting mental health, reducing stress, and enhancing cognitive functioning (54).

One of the notable advantages of MBIs is their efficacy in promoting psychological well-being and reducing stress and anxiety levels in a non-clinical population (56). Mindfulness techniques such as meditation, body scan, and mindful movement, enable individuals to cultivate heightened self-awareness, emotional regulation, and resilience. By adopting an accepting and non-judgmental stance towards their thoughts, emotions, and sensations, individuals foster a more constructive and adaptive relationship with their experiences (60). Consequently, individuals encounter enhancements in their overall psychological well-being, encompassing augmented levels of happiness, life satisfaction, and subjective well-being (61–63).

MBIs have demonstrated encouraging outcomes in managing various psychological and physical ailments in clinical populations. These interventions have proven successful in mitigating symptoms of depression, anxiety, stress, PTSD, and substance abuse disorders (55, 63–65). Additionally, MBIs have aided in managing chronic pain, elevating sleep quality, and augmenting immune system functioning (64, 66–73). MBIs equip individuals with useful techniques to navigate and cope with the challenges and difficulties related to their specific clinical circumstances by incorporating mindfulness practices into therapeutic interventions. These interventions encourage individuals to be more present and aware of their thoughts, emotions, and physical sensations, resulting in increased self-awareness and self-regulation. With consistent practice, MBIs have the potential to provide long-lasting benefits and improve overall well-being (74, 75).

It is also encouraging to note that we see similar benefits of MBIs in children. A review was recently conducted that included five studies assessing the effects of mindfulness-based interventions (MBIs) on anxiety in youth (76). The overall findings suggest that MBIs have positive and significant effects on anxiety disorders in youth. When individual study findings were examined, some interventions did not show significant effects, but when combined through meta-analysis, a significant pooled effect size was observed. These findings are consistent with prior reviews of MBIs in youth with various concerns.

## MBIs and surgery

To address the complexity and multidimensionality of surgery, researchers recommend that perioperative care should adopt a comprehensive approach that integrates non-pharmacological techniques, such as mindfulness, into standard surgical protocols (77). Mindfulness training has been increasingly recognized as an effective technique for helping patients manage preoperative anxiety and stress, with the potential to improve preoperative preparation, alleviate anxiety and stress, and enhance postoperative recovery (77–85). By cultivating present-moment awareness and non-judgmental acceptance of thoughts, feelings, and physical sensations, mindfulness practices empower patients to experience tranquillity, emotion regulation, resilience, and agency during surgical procedures.

Additionally, incorporating mindfulness-based practices into perioperative care may contribute to improved postoperative outcomes, including reduced complications, faster recovery, and enhanced psychological adjustment (77). Healthcare professionals can provide patients with invaluable tools for anxiety management, emotional well-being, and a more positive surgical experience by integrating mindfulness techniques into the perioperative care process. The practice of mindfulness, being non-invasive and accessible, holds great promise for optimizing patient care and promoting positive surgical experiences.

While mindfulness techniques have shown promising results in various healthcare settings, it is important to note that there is currently a gap in research concerning the application of mindfulness practices with children undergoing surgery. Despite the evidence supporting the benefits of mindfulness practice for adults in surgical contexts (reduced anxiety through increased emotion regulation), there is a lack of studies specifically examining its effectiveness and feasibility for pediatric surgical patients. This represents a crucial area that warrants further investigation to address the unique needs and challenges faced by children during the surgical process. Understanding the potential impact of mindfulness interventions on anxiety, distress, and overall well-being in pediatric surgery and the mechanisms behind the effects is essential for providing comprehensive and tailored care to young patients. Future research should aim to explore the benefits and feasibility of integrating mindfulness practice into the perioperative care of children, filling the current gap in knowledge and improving healthcare outcomes for this vulnerable population.

## Mindfulness and virtual reality

VR has emerged as a valuable tool for mindfulness-based interventions, particularly for individuals experiencing emotional dysregulation, by enhancing the central mechanisms of action in MBIs (86). Attention Regulation is an integral component of mindfulness. It involves maintaining focused attention on the current moment, separating mindfulness from other cognitive strategies. However, achieving this level of attention can be challenging in a world filled with distractions. VR technology, by creating immersive and controlled environments, minimizes these external distractions, offering a conducive space for individuals to concentrate more effectively on the present moment (54, 87). Body Awareness, another key aspect of mindfulness, involves developing an understanding and awareness of one's physical state. VR technology, especially when used in conjunction with biofeedback, can elevate this understanding. By providing real-time visual or auditory feedback based on physiological data such as heart rate or breathing rate, VR helps individuals form a closer connection between their mental states and physical responses, a crucial element in mindfulness (88).

The ability to manage and respond effectively to one's emotional experiences, known as Emotional Regulation, is also supported by VR. By creating controlled environments, VR allows individuals to face emotional triggers in a safe space. This practice, over time, can enhance emotional regulation skills, reducing overall emotional reactivity, a significant contributor to mental health and well-being (89). Another area positively impacted by VR is Self-Perception. Mindfulness aims to foster “decentering,” where one's thoughts and feelings are seen as transient events in the mind, not definitive aspects of their identity. VR experiences can simulate different perspectives, aiding individuals in understanding their thoughts and emotions as part of their conscious experience and not defining aspects of their selves. This can encourage a more objective, less judgmental self-view (86, 87, 89).

Lastly, the engagement factor in any intervention significantly determines its efficacy. Traditional mindfulness practices can feel monotonous to some individuals, potentially impacting their motivation. However, VR offers a dynamic and immersive experience, making mindfulness practice more appealing, leading to more consistent practice, which is crucial for reaping the benefits of MBIs (86, 87, 89).

Preliminary studies have shown positive results in improving adherence, effectiveness, and therapeutic outcomes in mindfulness practice, smoking cessation, chronic pain reduction, improved well-being, increased self-compassion and reduced anxiety (14, 86, 87, 89–94). Gamification strategies and bio/neurofeedback integration enhance engagement and customization (86, 87). However, further research is necessary to comprehend the relationship between VR and mindfulness and design optimal VR-supported mindfulness experiences.

While VR has been extensively used in exposure-based therapy, its application in mindfulness-based interventions is still evolving. Studies in non-clinical populations have demonstrated improvements in negative mood states, anxiety reduction, and positive outcomes in mindfulness skills, although the specific contributions of VR guidance and sensorial stimulation require further clarification (86). Neurophysiological measurements have been incorporated to assess brain activity and physiological responses, but standardization is lacking. Commercially available VR systems offer varying immersion levels and ease of use (86). Overcoming limitations, such as brief interventions, study design weaknesses, technological variability, demographic factors, and assessment measures, is crucial to establish the effectiveness of VR-mediated mindfulness interventions.

VR can enhance the feeling of being present in the moment through a sense of presence. It can also provide a sense of safety and novelty, benefiting individuals with anxiety or trauma-related disorders and increasing engagement and motivation (87). VR-mediated interventions address the limitations of traditional mindfulness interventions, offering accessibility, standardized environments, and flexibility for individuals with busy schedules or limited access to specialized programs (86, 87). However, challenges include the potential for cybersickness, cost barriers, and the need to monitor addiction to VR interventions.

## Mindfulness, virtual reality, & preoperative anxiety

At this time, no study has specifically examined mindfulness-based interventions delivered through VR exclusively for children to reduce preoperative anxiety. In fact, only one pre-registration study was found, in which they intend to investigate guided relaxation-based VR vs. distraction-based VR in reducing pediatric postoperative pain (95). As a secondary outcome, they will investigate its impact on perioperative anxiety. It hasn't even been studied in an adult surgical population yet.

Despite no study existing in this specific area, there are indications from the existing literature mentioned above that suggest potential benefits for using VR-based MBIs to alleviate preoperative anxiety in children. However, it is crucial to emphasize the need for empirical evidence to substantiate these claims. As a reminder, existing studies that have explored VR-based interventions in various populations, such as adults and non-clinical samples, have shown positive outcomes related to anxiety reduction, well-being enhancement, and improved adherence (14, 89, 91–94). These findings suggest that VR has the potential to be an effective tool for supporting the current need.

Given the potential benefits observed in other populations and considering the unique characteristics of VR and its compatibility with the mechanisms of mindfulness, it is reasonable to infer that VR-based MBIs may hold promise for reducing preoperative anxiety in children. VR offers an immersive and engaging experience that can capture children's attention and create a sense of presence, potentially facilitating their engagement in mindfulness practices and reducing anxiety associated with surgical procedures. Additionally, VR can provide a safe and controlled environment, which may alleviate fears and concerns commonly experienced by children undergoing surgery.

However, it is essential to underscore the importance of empirical research, specifically investigating the efficacy of VR-based MBIs in reducing preoperative anxiety in children. Rigorous studies are needed to examine the effects of this intervention approach on anxiety levels, psychological well-being, and other relevant outcomes in the pediatric population. Such research would provide valuable evidence to support the use of VR-based MBIs as a safe and effective intervention to mitigate preoperative anxiety in children, ultimately improving their surgical experience and overall well-being.

## Nature

The correlation between nature and mental health is a well-established and valued phenomenon in scientific research and societal norms. Numerous studies have consistently demonstrated the favourable impact of exposure to natural environments on psychological well-being (96, 97). Humans have understood the calming and restorative effects of nature for centuries, and contemporary scientific research is now able to quantify these benefits. There is evidence to support the notion that regular contact with nature can help in-part to alleviate a variety of mental health conditions, such as depression, anxiety, and stress disorders (96, 97). It is essential to recognize the significance of this relationship and to encourage the integration of nature-based interventions into mental health treatment plans.

Exposure to natural environments has been empirically associated with an array of health benefits, encompassing enhancement in cardiovascular and respiratory health, optimization of cognitive and immune function, and a reduced predisposition toward disease and mortality (98–100). The physical and visual engagement with natural surroundings has also been implicated in facilitating superior mental health outcomes. These engagements include but are not limited to: attenuation of stress, anxiety, and rumination, a diminution in somatization symptoms, and amelioration in biomarkers indicative of stress, cumulatively contributing to an elevated state of psychological well-being (98). Researchers have documented a relationship between exposure to natural settings and a decrement in cortisol levels, a hormone intricately entwined with stress responses (98). The multifaceted components of nature (auditory, visual, olfactory) synergistically work towards mitigating physiological responses to stress. Moreover, the inherent restorative characteristics of nature, offering opportunities for mental escapism and fascination, have been demonstrated to foster feelings of relaxation and provide relief from the omnipresent pressures inherent in contemporary lifestyles (98, 99). This interaction with the natural world, thus, functions as a potent countermeasure against stress.

Several theories have been developed to try and explain the link between nature and mental health. First, is the Biophilia Hypothesis, which was introduced by biologist E.O. Wilson in 1984. This theory posits that human beings possess an inherent inclination to connect with nature and other life forms (101, 102). This inclination can be traced back to human evolution, which occurred in close association with the natural environment (102). As a result, the Biophilia Hypothesis is believed to provide a fundamental basis for the correlation between exposure to natural surroundings and mental health outcomes (102, 103). The absence or scarcity of natural environments can result in cognitive exhaustion, stress, and other negative psychological effects. Conversely, as per this hypothesis, engagement with nature bolsters cognitive ability, enhances mood, reduces stress, and boosts overall well-being, thereby significantly impacting mental health (103).

It is widely acknowledged that the natural world is a source of inspiration and rejuvenation, and the Biophilia Hypothesis builds on this belief by arguing that humans have an innate connection to the natural world (103). This perspective has important implications for healthcare and public policy, as it suggests that access to green spaces and natural environments should be considered an essential aspect of human well-being. In addition, the Biophilia Hypothesis has been used to justify the use of biophilic design in various settings, such as hospitals, schools, and workplaces, which aim to incorporate elements of nature into the built environment (103).

Furthermore, the Biophilia Hypothesis has been used to explain the appeal of natural environments, such as parks, gardens, and forests, to individuals across cultures and age groups. It also provides a theoretical framework for the emerging field of ecotherapy, which uses exposure to natural environments as an adjunctive treatment for various mental health conditions (104, 105). The Biophilia Hypothesis has, therefore, been the subject of significant research and discussion, and has contributed to our understanding of the complex interplay between human beings and the natural world (106).

The Attention Restoration Theory (ART), proposed by Kaplan and Kaplan in the 1980s, posits that exposure to nature can help restore our attention capacities, thereby reducing mental fatigue and stress (107). This theory has important implications for mental health, as it suggests a mechanism by which natural environments can contribute to psychological well-being. According to ART, there are two types of attention: directed attention and involuntary attention. Directed attention, which is used in activities that require effort and concentration, can be depleted, leading to mental fatigue and stress. Conversely, involuntary attention, engaged effortlessly when captivated by inherently intriguing stimuli, allows directed attention to rest and recover when in natural environments (108).

Empirical research provides evidence supporting ART. Studies have shown that exposure to natural environments can lead to improved cognitive functioning, including attention restoration (109). These restorative effects of nature have been associated with improved mood, decreased stress levels, and better mental health outcomes overall. As such, ART offers a theoretical framework for understanding the restorative effects of nature on mental health and underscores the importance of access to green spaces for promoting mental health (110).

## Nature & surgery

The association between nature and surgical procedures is a complex interplay that considers the influence of interactions with natural surroundings on patient outcomes, both before and after surgery. Wilson's Biophilia Hypothesis holds relevance in a surgical context, as increasing evidence suggests that exposure to nature can have beneficial effects on surgical patients. Prior to surgery, exposure to natural environments may have stress-reducing effects that can enhance patient resilience and mental preparedness. Ulrich's Stress Reduction Theory (SRT) suggests that visual or physical access to nature can lead to emotional and physiological stress reduction (111). Moreover, some studies suggest that viewing natural scenes can result in reduced preoperative anxiety (112). This is significant as high preoperative anxiety levels have been associated with poorer postoperative outcomes.

After surgery, exposure to nature can support the recovery process. It is postulated that visual or physical interaction with natural settings can promote restorative effects, both mentally and physically. The Attention Restoration Theory (ART), developed by Kaplan and Kaplan, provides a theoretical framework for these effects, suggesting that exposure to nature helps restore our attentional capacities and reduces mental fatigue (107, 108). This could potentially support cognitive functioning and mood during the postoperative recovery phase. Moreover, a seminal study by Ulrich (111) found that patients recovering from surgery in rooms with views of nature had shorter hospital stays, took fewer analgesics, and had fewer postoperative complications compared to those with views of a brick wall. Such findings point towards the potential beneficial impacts of nature in the recovery process after surgery.

Furthermore, the concept of biophilic design—incorporating elements of nature into built environments—could be a promising approach to enhance patient access to nature within hospital settings, especially for those who cannot physically access outdoor environments post-surgery (113). Virtual reality technology also offers potential solutions, providing immersive natural experiences that may yield therapeutic benefits (114). While these perspectives underscore the potential benefits of nature exposure in the context of surgery, it is necessary to emphasize that more research is needed to understand the underlying mechanisms fully and to validate these effects across diverse patient populations and surgical contexts.

While the substantial benefits inherent in nature are widely acknowledged, surgery often leads to a period of hospitalization and/or immobilization, which can significantly impede an individual's access to natural environments. The immediate aftermath of surgery often imposes physical limitations on patients, confining them to their hospital beds and severely restricting their mobility (115). This restricted mobility, necessary for recuperation, could prevent patients from venturing outdoors and accessing natural environments. Furthermore, the necessity of maintaining a sterile environment in hospitals to prevent postoperative infections often limits patients' exposure to outdoor environments (115).

Additionally, the design and location of many hospitals might not facilitate regular access to natural environments. Many hospitals lack gardens or green spaces and may not even provide windows with views of nature (111, 116). These factors can further restrict patients' access to nature during their stay in the hospital. Moreover, time and resource constraints associated with postoperative care can also hinder access to nature. Patients' recovery processes often involve a rigorous schedule of medications, treatments, and possible rehabilitative activities, leaving little room for outdoor excursions. Additionally, staffing and resource constraints might prevent hospitals from facilitating such activities (117).

The presence of health conditions can considerably constrain an individual's access to nature, thereby limiting the therapeutic benefits that exposure to natural environments can offer. Such constraints arise due to a variety of factors. Firstly, many health conditions can lead to physical impairments or limitations that restrict mobility. For instance, chronic conditions such as arthritis or heart disease, or acute conditions requiring hospitalization, may limit a person's ability to move about freely and interact with nature (118). In cases of severe illnesses or conditions that require extended periods of bed rest, the barriers to accessing nature are particularly significant. Health conditions can also affect access to nature indirectly through the constraints imposed by treatment regimens. For example, individuals undergoing intensive medical treatments such as chemotherapy or dialysis may spend a significant amount of time in medical facilities, reducing their opportunities to engage with nature (117). Additionally, societal and infrastructural factors also play a role. In many urban environments, accessible green spaces may be scarce, which could disproportionately affect individuals with health conditions who may have limited mobility or resources (119). As a result, it is paramount to devise novel methodologies aimed at surmounting this ubiquitous challenge. Implementing novel strategies could guarantee that the recuperative essence of nature is not bound by any physical or health-related limitations, making it universally accessible.

## Nature & virtual reality

A literature review was recently conducted that in part, explored the use of virtual reality technology that incorporates natural settings (54). The authors highlight that VR technology has emerged as a proposed method for providing exposure to natural settings, particularly for those individuals who may not have access or are uncomfortable venturing into these environments (54, 120–123). This technology provides a feasible and cost-effective means of enabling realistic interactions with nature (16, 121, 122).

Growing evidence supports the therapeutic potential of 360-degree VR nature videos, demonstrating improvements in mood and stress reductions in healthy young and middle-aged adults, as well as university students (124–126). These positive psychological impacts such as improved mood levels have been objectively measured using parameters like electrodermal activity, heart rate variability, and standardized affect scales (124–126).

Beyond psychological well-being, VR nature experiences have been linked to enhancements in cognitive functioning (127, 128). Studies have suggested that immersive nature videos can restore attention and reduce physiological stress levels (17, 127). For instance, a study featuring 360-degree nature videos demonstrated restorative effects on attention, measured both subjectively and objectively (127). Another study reported significant stress reductions when participants were exposed to VR visuals of parks and forests, paired with congruent natural auditory and olfactory stimuli (17).

However, Sadowski and Khoury (54) point out that the current research on VR-based nature experiences comes with limitations. They highlight that most studies have focused on healthy, younger, or middle-aged adults, often with small sample sizes, thus restricting the generalizability of the findings (54). Also, while many studies have evaluated both physiological and psychological outcomes, the lack of longitudinal research leaves the long-term effects of VR nature experiences unclear. Consequently, more studies are needed to clarify the lasting impact of such interventions on affective states, cognitive function, and stress levels (54).

## Nature, virtual reality & preoperative anxiety

The literature search conducted did not yield any studies explicitly investigating the application of nature-based virtual reality (VR) interventions to alleviate preoperative anxiety in children. Moreover, no such studies were found targeting an adult population. Nevertheless, the existing body of literature implies the potential advantages of employing nature-based VR experiences to attenuate preoperative anxiety in children. Several studies have probed the use of VR interventions across varied demographics, including adults and non-clinical samples, reporting positive effects in terms of reducing anxiety and enhancing overall well-being (54).

Virtual reality interventions, particularly those incorporating natural environments, have the potential to provide an engrossing and captivating experience. This could be particularly beneficial in engaging children and reducing anxiety associated with surgical procedures. Additionally, VR presents a secure and controlled setting that could alleviate the typical fears and apprehensions associated with surgical procedures in pediatric patients. However, it is paramount to emphasize the necessity for rigorous empirical research specifically evaluating the effectiveness of nature-based VR interventions in managing preoperative anxiety in children. Comprehensive studies are warranted to assess the impact of such interventions on anxiety levels, psychological well-being, and other relevant outcomes in the pediatric demographic. This would provide compelling evidence for the application of nature-based VR interventions as a safe and efficacious approach to managing preoperative anxiety in children, thereby enhancing their surgical experience and overall well-being.

## Mindfulness, nature & VR: complementary tools to reduce preoperative anxiety

The connection between mindfulness and nature lies in their shared ability to induce a sense of tranquility and mental clarity, and a deeper connection with oneself and the natural world, ultimately supporting the key mechanisms of mindfulness. Practicing mindfulness in a natural environment facilitates attention regulation by anchoring the mind in present-moment experiences (11). It encourages body awareness as individuals attune to their physical responses to the natural world. It also aids emotional regulation, as being in nature often provokes feelings of peace and contentment, providing a serene backdrop for observing and accepting emotions (129). Lastly, immersing oneself in the natural environment can encourage a shift in self-perception, promoting a sense of interconnectedness and minimizing ego-centric thinking, aligning with the “decentering” goal of mindfulness (130).

Virtual reality (VR) technology offers exciting possibilities for combining mindfulness and nature experiences, providing immersive and realistic environments that can enhance the effectiveness of therapeutic interventions, particularly through the mechanisms of mindfulness (88). VR allows individuals to be transported to simulated natural settings, where they can engage in mindfulness practices, facilitating attention regulation by minimizing external distractions and focusing on the present moment within the natural environment (14). This immersive technology also helps improve body awareness, as individuals can attune to their physical responses to the virtually represented elements of nature. Emotional regulation is further facilitated, as VR can realistically depict serene environments such as forests or beaches, triggering peaceful and calming emotions that can help in processing emotional experiences effectively.

In the specific context of pediatric preoperative anxiety, the combination of mindfulness, nature, and VR can aid in fostering a sense of decentering. By creating virtual environments that depict serene natural landscapes, children can gain perspective that their anxious thoughts and feelings are transient and do not define their experience (13). Within these virtual environments, children can engage in guided mindfulness exercises tailored to their age and cognitive abilities, such as focused breathing or body scan meditation (63). These exercises, when paired with the engaging and immersive VR nature environment, can significantly enhance their mindfulness practice. It not only makes the practice more appealing, thereby encouraging consistency, but it also strengthens their ability to regulate emotions and maintain attention, contributing to a reduction in preoperative anxiety.

To ensure the effectiveness of VR-based mindfulness in nature interventions for pediatric preoperative anxiety, comprehensive research is warranted. Rigorous studies should explore various aspects, such as the optimal design of VR environments, the specific types and durations of mindfulness exercises, and the most effective ways to introduce and support children in using these interventions. It is crucial to consider factors such as age-appropriate content, engaging interactive elements, and tailored guidance to facilitate a positive and impactful experience for children.

Furthermore, research should examine the underlying mechanisms through which mindfulness in nature and VR-based interventions exert their effects on preoperative anxiety. This could involve investigating physiological markers of stress, neural activity patterns, and subjective experiences reported by children during and after the VR interventions. Long-term follow-up studies are also essential to evaluate the durability of the intervention's effects and assess its potential for fostering long-lasting resilience and well-being in children facing surgery.

By integrating mindfulness, the healing power of nature, and the immersive qualities of VR technology, interventions targeting pediatric preoperative anxiety can be comprehensive, engaging, and tailored to meet the unique needs of children. As the field continues to evolve, ongoing research and innovation will further advance our understanding and refine the use of mindfulness in nature delivered through VR as a valuable intervention tool for reducing pediatric preoperative anxiety. A summary of findings can be found in Table 1.

**Table 1:** Mindfulness-based interventions in pediatric surgical practice.

Aspect	Description
Prevalence of preoperative anxiety	Over 75% of pediatric surgery patients experience preoperative anxiety, leading to complicated recoveries.
Physiological manifestations of anxiety	Symptoms include restlessness, labored breathing, trembling, crying, and in some cases, physical restraint is required.
Current interventions	Existing interventions (e.g., puppet shows, animators) are less effective for older children and lack self-regulation techniques.
Mindfulness-based interventions (MBIs)	MBIs involve purposeful, non-judgmental attention to the present moment and can effectively reduce mental health symptoms in youth.
Challenges in practicing mindfulness	Children may find practicing mindfulness challenging and boring due to the lack of sensory input and engagement.
Role of Virtual Reality (VR) in MBIs	VR provides an engaging 3-D practice environment, minimizing distractions and enhancing presence, making MBIs more appealing and effective for children.
Nature-based VR settings	Nature-based VR settings in mindfulness training can bridge the gap for beginners by providing an engaging, distraction-minimized environment that enhances presence.

## Critical evaluation: limitations, gaps, future directions

Limitations of a nature-based mindfulness virtual reality intervention to reduce pediatric preoperative anxiety can be identified in several areas. Firstly, there is limited research specifically focusing on the use of nature-based mindfulness virtual reality interventions for pediatric preoperative anxiety. The scarcity of studies restricts the depth of understanding regarding its effectiveness and specific application in this context (43). While studies have demonstrated the effectiveness of virtual reality interventions in reducing anxiety in children, particularly in the preoperative setting, there is a need for more research specifically examining the impact of nature-based mindfulness interventions.

Additionally, the heterogeneity of interventions used in studies poses a challenge in drawing definitive conclusions about the most effective elements of the intervention. Nature-based mindfulness virtual reality interventions may vary in terms of content, duration, and specific components, which can impact their effectiveness (131). Establishing standardized protocols for implementing nature-based mindfulness virtual reality interventions is another critical gap in the literature. This includes determining the optimal duration, frequency, and content of intervention sessions, as well as identifying specific nature elements and mindfulness techniques that are most effective in reducing pediatric preoperative anxiety.

Moreover, many studies investigating a form of this intervention often have small sample sizes, limiting the generalizability of the findings to a larger population. The bulk of the research that closely relates to this review was conducted in Asia, further impacting the generalizability of the results. While a lot of research has been conducted with adults, it is not appropriate to make assumptions or extrapolate findings to the youth population without specific evidence. Future research should focus on youth-specific interventions and consider the unique characteristics and needs of this population. The lack of long-term follow-up is another limitation. Many studies focus on short-term outcomes immediately before or after the surgical procedure (one time post intervention follow up only), but the long-term effects (3–6 months post intervention) of a nature-based mindfulness virtual reality intervention on sustained anxiety reduction and postoperative recovery are not well-explored. It is crucial to investigate the sustained impact of these interventions on postoperative anxiety, recovery, and overall well-being.

The external validity of the findings may be limited due to the controlled research settings in which studies are conducted. Real-world clinical settings may present different challenges, patient characteristics, and dynamics that could affect the effectiveness of the intervention. Furthermore, comparative studies directly comparing the nature-based mindfulness VR intervention with other standard anxiety management techniques or alternative interventions are lacking. Comparative studies are necessary to determine the relative efficacy and advantages of the intervention compared to existing approaches. Conducting such studies would provide valuable insights into the relative benefits and potential synergies of different approaches, including pharmacological and traditional non-pharmacological interventions.

Moreover, while initial studies have shown promise, the long-term feasibility and scalability of implementing nature-based mindfulness VR interventions in real-world clinical settings are yet to be fully understood. Further research should explore the practical considerations, implementation challenges, and potential integration of these interventions into existing healthcare workflows. Addressing these gaps through rigorous research will contribute to the advancement of knowledge and application of nature-based mindfulness VR interventions for reducing pediatric preoperative anxiety, ultimately enhancing perioperative care and postoperative outcomes for children.

Advancements in VR technology should be considered to ensure the continued effectiveness and engagement of Nature-Based Mindfulness Programs using VR. Staying up-to-date with improvements in visual and auditory fidelity, immersive experience, and user-friendly interfaces of VR platforms can enhance the effectiveness and acceptability of these interventions.

Addressing these limitations through further research, larger and more diverse samples, long-term follow-ups, rigorous study designs, and consideration of ethical and practical implementation factors will contribute to a better understanding of the effectiveness and potential of nature-based mindfulness VR interventions for reducing pediatric preoperative anxiety. By addressing these future directions, researchers and practitioners can optimize Nature-Based Mindfulness Programs using VR to effectively reduce pediatric perioperative anxiety and improve outcomes for children undergoing surgery.

## Implications

### Implications for research

The utilization of nature-based mindfulness programs in VR to alleviate pediatric perioperative anxiety introduces numerous research implications, each bearing significance for the future of perioperative care and the development of non-pharmacological interventions. The first major implication centers on establishing the efficacy and effectiveness of these interventions. Thorough investigation through well-structured Randomized Controlled Trials (RCTs) is imperative to substantiate the value of VR-based interventions compared to traditional methods of treatment or control conditions. The impact of these interventions must be gauged not solely by the degree to which they reduce anxiety, but also by their potential to enhance other facets of the patient experience. This includes measuring the effects on pain perception, patient satisfaction, recovery times, and overall healthcare experience. These studies will be foundational in developing a persuasive evidence base for the adoption of such technologies in routine pediatric perioperative care.

In tandem with understanding the overall effectiveness of these interventions, it is also crucial to unearth the mechanisms of action that underpin their utility. This involves in-depth examination of the components that make VR-based nature mindfulness programs effective at reducing perioperative anxiety. Are the therapeutic benefits driven by immersion, engagement, distraction, relaxation, or the mindfulness practices themselves? Detailed exploration into these questions can provide valuable insights for fine-tuning these interventions and enhancing their therapeutic potential.

Another significant research implication lies in the personalization and optimization of these interventions for individual patients. This could involve understanding how different virtual environments or mindfulness practices influence the effectiveness of the program, as well as how much guidance or interaction should be provided. Investigations in this area can further our understanding of how to tailor VR-based mindfulness interventions to cater to the unique needs and preferences of each child, which could significantly enhance the effectiveness and acceptability of these interventions.

The long-term effects of these interventions represent another important area for research. Longitudinal studies can shed light on whether these interventions lead to sustained reductions in anxiety, foster improved resilience, or develop better coping skills in the postoperative period. Such findings could have far-reaching implications for pediatric perioperative care, and potentially for the broader field of pediatric health care.

There's also a significant need to understand the practicalities and logistics of implementing these interventions. This includes studying the cost-effectiveness of VR-based nature mindfulness programs, understanding how they can be seamlessly integrated into existing healthcare workflows, and identifying the training needs of healthcare providers who will deliver these interventions. The findings from this line of research can inform strategies to scale up these interventions and ensure their sustainable adoption within healthcare settings.

Equally important is the need to research issues around accessibility and equity. Given the technological nature of VR-based mindfulness interventions, there's potential for disparities in access due to factors such as socioeconomic status, geographical location, or sensory impairments. Uncovering these barriers and devising strategies to overcome them is essential to ensure that all pediatric patients can benefit from these interventions, irrespective of their background or circumstances.

Finally, it is of paramount importance to ensure that these interventions are safe and acceptable for pediatric patients. This necessitates research into potential side effects of VR usage, such as motion sickness or disorientation, and the investigation into patients’ and parents' attitudes towards these interventions. Establishing the safety and acceptability of these interventions is a key step towards their wider acceptance and use in pediatric perioperative care.

In summary, while the potential of nature-based mindfulness programs using VR in reducing pediatric perioperative anxiety is significant, there is a pressing need for rigorous research to unlock their full potential. This research should focus on a broad range of areas, from effectiveness and mechanisms of action to practical and ethical considerations, in order to provide a comprehensive understanding of how best to utilize these innovative interventions in pediatric perioperative care.

### Implications for practice

Implementing a mindfulness program based on nature and utilizing VR can be an effective approach to reducing pediatric perioperative anxiety. This could have significant clinical implications, including improving the overall patient experience. Traditional strategies such as sedation can have unwanted side effects, and a non-pharmacological intervention like the VR program can provide a valuable alternative to manage anxiety in pediatric patients.

Another important implication of the program is the potential for direct impacts on medical outcomes. High levels of anxiety can impact physiological responses to stress, which can lead to complications during and after surgery. By reducing anxiety, this VR program could improve perioperative outcomes and potentially reduce recovery time. Moreover, if children find the VR experience enjoyable, they may be more compliant with medical procedures and preoperative instructions, leading to smoother procedures and potentially better outcomes.

However, implementing such a program would require significant resources. Healthcare providers like clinicians and nurses would need training to implement the VR program correctly and troubleshoot any technical issues. They would also need to learn how to integrate the program into their current workflows without causing disruptions. This could pose a significant challenge, particularly in busy perioperative environments.

Another potential issue relates to healthcare costs. While the VR program could potentially reduce healthcare costs in the long run by improving outcomes and reducing complications, the upfront cost of implementing the program (including equipment, software, and training) could be substantial. Thus, cost-effectiveness analyses would be necessary to determine the program's financial viability.

Moreover, clinicians would also need to consider patient selection. While VR offer innovative ways to administer MBIs for pediatric patients, there are notable limitations that make these technologies less amenable for some children, including age restrictions and seizure thresholds. Younger children, especially those under the age of 10, may find it challenging to use VR due to the size and weight of the headsets, and the complexity of the technology. Additionally, concerns regarding the impact of VR on the developing brains of younger children necessitate cautious use. Children with a history of seizures or those at risk of seizures must be carefully considered, as the flashing lights and rapidly changing visuals in VR environments can trigger seizures in susceptible individuals. Clinicians would need guidelines to help them determine who can safely and effectively use the VR program.

When looking at the broader picture, if successful, these types of interventions could lead to new standards of care in perioperative management for children. This could result in widespread adoption and potential changes in policies and guidelines. However, this also raises questions about equity in healthcare. Not all healthcare settings may be able to afford the technology required for VR, which could lead to disparities in who has access to these potentially beneficial interventions.

Given these constraints, it's crucial to explore alternative methods to apply nature-based mindfulness processes without relying on headsets. One approach is the use of immersive natural environments in controlled physical spaces, designed to replicate natural settings with elements like indoor plants, nature sounds, and panoramic visuals of natural scenes. Another alternative is guided nature walks or outdoor mindfulness sessions, leveraging the calming effects of nature and effective for children who benefit from physical movement and direct interaction with the environment. Acknowledging these limitations of VR is essential in developing diverse approaches that cater to the unique needs and conditions of young patients.

In summary, introducing a VR-based nature mindfulness program into pediatric perioperative care could bring about a paradigm shift in the management of perioperative anxiety, improve patient outcomes, and enhance the overall experience. Nonetheless, it's important to consider the costs, training requirements, patient selection criteria, and potential for inequity. Further research is needed to validate its effectiveness and evaluate its practicality in diverse healthcare settings. As a result, this innovative approach could be a valuable and promising addition to the perioperative care of pediatric patients, but its implementation requires careful consideration of its clinical, practical, and ethical implications.

## Conclusion

In conclusion, this review has examined the risks associated with anxiety in children undergoing surgery, emphasizing its impact on physical recovery. It has also explored the use of virtual reality as an intervention to reduce anxiety in children, providing an overview of the current VR interventions employed in hospitals. Furthermore, the review has explored the use of VR in relation to nature and mindfulness separately, discussing theoretical conceptualizations, clinical applications, and potential therapeutic efficacy. By examining the connection between mindfulness, nature, and VR, this review has explored the potential for an effective intervention. The literature supporting this connection has been synthesized, highlighting its relevance. Additionally, the review has addressed the limitations, findings, gaps, and contradictions in the existing literature, providing valuable insights. Despite the limitations of the current research, this review contributes to the existing knowledge and concludes with research and clinical implications for further exploration and application of VR interventions in the context of anxiety management in children undergoing surgery.

Implementing a VR-based nature mindfulness program may have the potential to effectively reduce pediatric perioperative anxiety, improving patient experience and potentially enhancing medical outcomes. However, challenges such as resource requirements, cost-effectiveness, patient selection, and equity need to be considered. Further research is necessary to explore its effectiveness and practicality. Overall, this innovative approach holds promise for improving perioperative care in children.

## References

[1] National Cancer Institute. Definition of perioperative—NCI Dictionary of Cancer Terms—NCI (nciglobal,ncienterprise) [NciAppModulePage]. (2011). Available at: https://www.cancer.gov/publications/dictionaries/cancer-terms/def/perioperative

[2] Gulur P Fortier MA Mayes LC Kain ZN. 3—perioperative Behavioral stress in children. In Coté C Lerman J Anderson BJ, editors. A Practice of Anesthesia for Infants and Children, 6th ed. North York: Elsevier (2019). p. 25–34.e3. 10.1016/B978-0-323-42974-0.00003-3

[3] Wright KD Stewart SH Finley GA Buffett-Jerrott SE. Prevention and intervention strategies to alleviate preoperative anxiety in children: a critical review. Behav Modif. (2007) 31(1):52–79. 10.1177/0145445506295055 17179531

[4] Fronk E Billick SB. Pre-operative anxiety in pediatric surgery patients: multiple case study analysis with literature review. Psychiatric Quarterly. (2020) 91(4):1439–51. 10.1007/s11126-020-09780-z 32424544

[5] Costa Fernandes S Arriaga P. The effects of clown intervention on worries and emotional responses in children undergoing surgery. J Health Psychol. (2010) 15(3):405–15. 10.1177/1359105309350231 20348361

[6] Turgoose DP Kerr S Coppi PD Blackburn S Wilkinson S Rooney N Prevalence of traumatic psychological stress reactions in children and parents following paediatric surgery: a systematic review and meta-analysis. BMJ Paediatrics Open. (2021) 5(1):e001147. 10.1136/bmjpo-2021-001147 34337164 PMC8287603

[7] Kain ZN Mayes LC Caldwell-Andrews AA Karas DE McClain BC. Preoperative anxiety, postoperative pain, and behavioral recovery in young children undergoing surgery. Pediatrics. (2006) 118(2):651–8. 10.1542/peds.2005-2920 16882820

[8] Vaezzadeh N Douki ZE Hadipour A Osia S Shahmohammadi S Sadeghi R. The effect of performing preoperative preparation program on school age children’s anxiety. Iran J Pediatr. (2011) 21(4):6. PMC3446145 23056832

[9] Lerwick JL. Psychosocial implications of pediatric surgical hospitalization. Semin Pediatr Surg. (2013) 22(3):129–33. 10.1053/j.sempedsurg.2013.04.003 23870205

[10] Li WHC Chung JOK Ho KY Kwok BMC. Play interventions to reduce anxiety and negative emotions in hospitalized children. BMC Pediatr. (2016) 16(1):36. 10.1186/s12887-016-0570-5 26969158 PMC4787017

[11] Kabat-Zinn J. Mindfulness-based interventions in context: past, present, and future. Clin Psychol (New York). (2006) 10(2):144–56. 10.1093/clipsy.bpg016

[12] Hendry CF Breitmeyer AM. The complexities of youth depression: the potential of mindfulness- and compassion-based interventions. J Child Adolesc Counsel. (2022) 8(1):46–58. 10.1080/23727810.2022.2040318

[13] Li BJ Bailenson JN Pines A Greenleaf WJ Williams LM. A public database of immersive VR videos with corresponding ratings of arousal, valence, and correlations between head movements and self report measures. Front Psychol. (2017) 8. 10.3389/fpsyg.2017.02116 PMC5723428 29259571

[14] Navarro-Haro MV López-Del-Hoyo Y Campos D Linehan MM Hoffman HG García-Palacios A Meditation experts try virtual reality mindfulness: a pilot study evaluation of the feasibility and acceptability of virtual reality to facilitate mindfulness practice in people attending a mindfulness conference. PloS One. (2017) 12(11):e0187777. 10.1371/journal.pone.0187777 29166665 PMC5699841

[15] Robertson CE Hermann KL Mynick A Kravitz DJ Kanwisher N. Neural representations integrate the current field of view with the remembered 360° panorama in scene-selective Cortex. Curr Biol. (2016) 26(18):2463–8. 10.1016/j.cub.2016.07.002 27618266

[16] Browning MHEM Mimnaugh KJ van Riper CJ Laurent HK LaValle SM. Can simulated nature support mental health? Comparing short, single-doses of 360-degree nature videos in virtual reality with the outdoors. Front Psychol. (2019) 10:2667. 10.3389/fpsyg.2019.02667 32010003 PMC6974516

[17] Hedblom M Gunnarsson B Iravani B Knez I Schaefer M Thorsson P Reduction of physiological stress by urban green space in a multisensory virtual experiment. Sci Rep. (2019) 9(1):10113. 10.1038/s41598-019-46099-7 31300656 PMC6625985

[18] Jerdan SW Grindle M van Woerden HC Kamel Boulos MN. Head-mounted virtual reality and mental health: critical review of current research. JMIR Serious Games. (2018) 6(3):e14. 10.2196/games.9226 29980500 PMC6054705

[19] Yu C-P Lee H-Y Lu W-H Huang Y-C Browning MHEM. Restorative effects of virtual natural settings on middle-aged and elderly adults. Urban For Urban Green. (2020) 56:126863. 10.1016/j.ufug.2020.126863

[20] Chow CHT Van Lieshout RJ Buckley N Schmidt LA. Children’s perioperative multidimensional anxiety scale (CPMAS): development and validation. Psychol Assess. (2016) 28(9):1101–9. APA PsycInfo <2016>. 10.1037/pas0000318 27537004

[21] Watson AT Visram A. Children’s preoperative anxiety and postoperative behaviour. Pediatric Anesthesia. (2003) 13(3):188–204. 10.1046/j.1460-9592.2003.00848.x 12641680

[22] Sebastian Q Carrillo FX Ortigosa J. Pre-surgical worries: an empirical study in the child and adolescent population. Anales Españoles de Pediatría. (2001) 55:129–34. 10.1016/S1695-4033(01)77648-5 11472664

[23] Kain ZN. Postoperative maladaptive behavioral changes in children: incidence, risks factors and interventions. Acta Anaesthesiol Belg. (2000) 51(4):217–26. 11129622

[24] Kain ZN Mayes LC O’Connor TZ Cicchetti DV. Preoperative anxiety in children. Predictors and outcomes. Arch Pediatr Adolesc Med. (1996) 150(12):1238–45. 10.1001/archpedi.1996.02170370016002 8953995

[25] Kotiniemi LH Ryhänen PT Moilanen IK. Behavioural changes in children following day-case surgery: a 4-week follow-up of 551 children. Anaesthesia. (1997) 52(10):970–6. 10.1111/j.1365-2044.1997.202-az0337.x 9370839

[26] Thompson ML. Information-seeking coping and anxiety in school-age children anticipating surgery. Children’s Health Care. (1994) 23(2):87–97. 10.1207/s15326888chc2302_2 10171872

[27] Tiedeman ME Clatworthy S. Anxiety responses of 5- to 11-year-old children during and after hospitalization. J Pediatr Nurs. (1990) 5(5):334–43. 10.5555/uri:pii:088259639090005T 2213477

[28] Vetter TR. The epidemiology and selective identification of children at risk for preoperative anxiety reactions. Anesth Analg. (1993) 77(1):96–9. 10.1213/00000539-199307000-00019 8317755

[29] Hägglöf B. Psychological reaction by children of various ages to hospital care and invasive procedures. Acta Paediatrica. (1999) 88(431):72–8. 10.1111/j.1651-2227.1999.tb01321.x 10588274

[30] Kotiniemi LH Ryhänen PT Moilanen IK. Behavioural changes following routine ENT operations in two-to-ten-year-old children. Paediatr Anaesth. (1996) 6(1):45–9. 10.1111/j.1460-9592.1996.tb00352.x 8839088

[31] McCann ME Kain ZN. The management of preoperative anxiety in children: an update. Anesth Analg. (2001) 93(1):98–105. 10.1097/00000539-200107000-00022 11429348

[32] McGraw T. Preparing children for the operating room: psychological issues. Can J Anaesth. (1994) 41(11):1094–103. 10.1007/BF03015661 7828259

[33] Koroluk LD. Dental anxiety in adolescents with a history of childhood dental sedation. ASDC J Dent Child. (2000) 67(3):200–5, 161. 10902080

[34] Pottinger AM Ehikhametalor O. Children’s response to hospitalization at the university hospital of the West Indies. West Indian Med J. (2000) 49(1):47–51. 10786452

[35] Blount RL Zempsky WT Jaaniste T Evans S Cohen LL Devine KA Management of Pediatric Pain and Distress due to Medical Procedures. In: Handbook of Pediatric Psychology, 4th ed. *American Academy of Pediatrics Committee on Drugs. (2002). Guidelines for monitoring and management of pediatric patients during and after sedation for diagnostic and therapeutic procedures: Addendum. Pediatrics, 110, 836-838*. New York: Jessica Stone (2009) p. 171–88. APA PsycInfo <2009>.

[36] Fischer S Vinall J Pavlova M Graham S Jordan A Chorney J Role of anxiety in young children’s pain memory development after surgery. Pain. (2019) 160(4):965–72. APA PsycInfo <2019>. 10.1097/j.pain.0000000000001473 30586022

[37] Gabriel MG Wakefield CE Vetsch J Karpelowsky JS Darlington A-SE Cohn RJ Paediatric surgery for childhood cancer: lasting experiences and needs of children and parents. Eur J Cancer Care (Engl). (2019) 28(5). APA PsycInfo <2019>. 10.1111/ecc.13116 31184790

[38] Rabbitts JA Aaron RV Fisher E Lang EA Bridgwater C Tai GG Long-term pain and recovery after major pediatric surgery: a qualitative study with teens, parents, and perioperative care providers. J Pain. (2017) 18(7):778–86. APA PsycInfo <2017>. 10.1016/j.jpain.2017.02.423 28232147 PMC5484738

[39] Ben-Amitay G Kosov I Reiss A Toren P Yoran-Hegesh R Kotler M Is elective surgery traumatic for children and their parents? J Paediatr Child Health. (2006) 42(10):618–24. APA PsycInfo <2006 to 2007>. 10.1111/j.1440-1754.2006.00938.x 16972969

[40] Addab S Hamdy R Thorstad K Le May S Tsimicalis A. Use of virtual reality in managing paediatric procedural pain and anxiety: an integrative literature review. J Clin Nurs. (2022) 31(21–22):3032–59. 10.1111/jocn.16217 35068011

[41] Burns-Nader S. Technological tools for supporting pediatric patients through procedures. In: Integrating Technology into Modern Therapies: A Clinician’s Guide to Developments and Interventions. New York: Routledge (2019). p. 181–93. APA PsycInfo <2019>.

[42] Dehghan F Jalali R Bashiri H. The effect of virtual reality technology on preoperative anxiety in children: a solomon four-group randomized clinical trial. Perioper Med. (2019) 8(1):5. 10.1186/s13741-019-0116-0 PMC6549331 31171963

[43] Eijlers R Dierckx B Staals LM Berghmans JM van der Schroeff MP Strabbing EM Virtual reality exposure before elective day care surgery to reduce anxiety and pain in children: a randomised controlled trial. Eur J Anaesthesiol. (2019) 36(10):728. 10.1097/EJA.0000000000001059 31356373 PMC6738544

[44] Gold JI Annick ET Lane AS Ho K Marty RT Espinoza JC. “Doc McsSuffins: doctor for a day” virtual reality (DocVR) for pediatric preoperative anxiety and satisfaction: pediatric medical technology feasibility study. J Med Internet Res. (2021) 23(4). APA PsycInfo <2021>. 10.2196/25504 PMC8094020 33730687

[45] Jung MJ Libaw JS Ma K Whitlock EL Feiner JR Sinskey JL. Pediatric distraction on induction of anesthesia with virtual reality and perioperative anxiolysis: a randomized controlled trial. Anesth Analg. (2021) 132(3):798. 10.1213/ANE.0000000000005004 32618627 PMC9387568

[46] Park J-W Nahm FS Kim J-H Jeon Y-T Ryu J-H Han S-H. The effect of mirroring display of virtual reality tour of the operating theatre on preoperative anxiety: a randomized controlled trial. IEEE J Biomed Health Inform. (2019) 23(6):2655–60. 10.1109/JBHI.2019.2892485 30640637

[47] Ryu J-H Park S-J Park J-W Kim J-W Yoo H-J Kim T-W Randomized clinical trial of immersive virtual reality tour of the operating theatre in children before anaesthesia. Br J Surg. (2017) 104(12):1628–33. 10.1002/bjs.10684 28975600

[48] Ryu J-H Park J-W Nahm FS Jeon Y-T Oh A-Y Lee HJ The effect of gamification through a virtual reality on preoperative anxiety in pediatric patients undergoing general anesthesia: a prospective, randomized, and controlled trial. J Clin Med. (2018) 7(9):9. 10.3390/jcm7090284 30227602 PMC6162739

[49] Ryu J-H Oh A-Y Yoo H-J Kim J-H Park J-W Han S-H. The effect of an immersive virtual reality tour of the operating theater on emergence delirium in children undergoing general anesthesia: a randomized controlled trial. Pediatric Anesthesia. (2019) 29(1):98–105. 10.1111/pan.13535 30365231

[50] Eijlers R Utens EMWJ Staals LM de Nijs PFA Berghmans JM Wijnen RMH Systematic review and meta-analysis of virtual reality in pediatrics: effects on pain and anxiety. Anesth Analg. (2019) 129(5):1344–53. 10.1213/ANE.0000000000004165 31136330 PMC6791566

[51] Simonetti V Tomietto M Comparcini D Vankova N Marcelli S Cicolini G. Effectiveness of virtual reality in the management of paediatric anxiety during the peri-operative period: a systematic review and meta-analysis. Int J Nurs Stud. (2022) 125. 10.1016/j.ijnurstu.2021.104115 34781118

[52] Bernaerts S Bonroy B Daems J Sels R Struyf D Gies I Virtual reality for distraction and relaxation in a pediatric hospital setting: an interventional study with a mixed-methods design. Front Digit Health. (2022) 4:866119. 10.3389/fdgth.2022.866119 35712230 PMC9192964

[53] Tennant M McGillivray J Youssef GJ McCarthy MC Clark T-J. Feasibility, acceptability, and clinical implementation of an immersive virtual reality intervention to address psychological well-being in children and adolescents with cancer. J Pediatr Oncol Nurs. (2020) 37(4):265–77. 10.1177/1043454220917859 32536320

[54] Sadowski I Khoury B. Nature-based mindfulness-compassion programs using virtual reality for older adults: a narrative literature review. Front Virtual Reality. (2022) 3. 10.3389/frvir.2022.892905

[55] Hofmann SG Sawyer AT Witt AA Oh D. The effect of mindfulness-based therapy on anxiety and depression: a meta-analytic review. J Consult Clin Psychol. (2010) 78(2):169–83. 10.1037/a0018555 20350028 PMC2848393

[56] Hofmann SG Gómez AF. Mindfulness-based interventions for anxiety and depression. Psychiatr Clin North Am. (2017) 40(4):739–49. 10.1016/j.psc.2017.08.008 29080597 PMC5679245

[57] Kabat-Zinn J. Wherever You Go, There You Are: Mindfulness Meditation in Everyday Life: Jon Kabat-Zinn: 9781401307783: Books—Amazon.ca. (2013). Available at: https://www.amazon.ca/Wherever-You-There-Are-Mindfulness/dp/1401307787

[58] Khoury B Knäuper B Pagnini F Trent N Chiesa A Carrière K. Embodied mindfulness. Mindfulness (N Y). (2017) 8(5):1160–71. 10.1007/s12671-017-0700-7

[59] Khoury B. Mindfulness: embodied and embedded. Mindfulness (N Y). (2018) 9(4):1037–42. 10.1007/s12671-017-0858-z

[60] Cheung RYM Lau EN-S. Is mindfulness linked to life satisfaction? Testing savoring positive experiences and gratitude as mediators. Front Psychol. (2021) 12. 10.3389/fpsyg.2021.591103 PMC7965977 33746824

[61] Brown KW Ryan RM. The benefits of being present: mindfulness and its role in psychological well-being. J Pers Soc Psychol. (2003) 84(4):822–48. 10.1037/0022-3514.84.4.822 12703651

[62] Coffey KA Hartman M. Mechanisms of action in the inverse relationship between mindfulness and psychological distress. Complement Health Pract Rev. (2008) 13(2):79–91. 10.1177/1533210108316307

[63] Garland EL Farb NA Goldin P Fredrickson BL. Mindfulness broadens awareness and builds eudaimonic meaning: a process model of mindful positive emotion regulation. Psychol Inq. (2015) 26(4):293–314. 10.1080/1047840X.2015.1064294 27087765 PMC4826727

[64] Goyal M Singh S Sibinga EMS Gould NF Rowland-Seymour A Sharma R Meditation programs for psychological stress and well-being: a systematic review and meta-analysis. JAMA Intern Med. (2014) 174(3):357–68. 10.1001/jamainternmed.2013.13018 24395196 PMC4142584

[65] Khoury B Lecomte T Fortin G Masse M Therien P Bouchard V Mindfulness-based therapy: a comprehensive meta-analysis. Clin Psychol Rev. (2013) 33(6):763–71. 10.1016/j.cpr.2013.05.005 23796855

[66] Gotink RA Chu P Busschbach JJV Benson H Fricchione GL Hunink MGM. Standardised mindfulness-based interventions in healthcare: an overview of systematic reviews and meta-analyses of RCTs. PLoS One. (2015) 10(4):e0124344. 10.1371/journal.pone.0124344 25881019 PMC4400080

[67] Kabat-Zinn J Lipworth L Burney R Sellers W. Four-year follow-up of a meditation-based program for the self-regulation of chronic pain: treatment outcomes and compliance. Clin J Pain. (1987) 3(1):60. 10.1097/00002508-198703010-00010

[68] Kabat-Zinn J Wheeler E Light T Skillings A Scharf MJ Cropley TG Influence of a mindfulness meditation-based stress reduction intervention on rates of skin clearing in patients with moderate to severe psoriasis undergoing photo therapy (UVB) and photochemotherapy (PUVA). Psychosom Med. (1998) 60(5):625. 10.1097/00006842-199809000-00020 9773769

[69] Rosenzweig S Reibel DK Greeson JM Edman JS Jasser SA McMearty KD Mindfulness-based stress reduction is associated with improved glycemic control in type 2 diabetes mellitus: a pilot study. Altern Ther Health Med. (2007) 13(5):36–8. 17900040

[70] Smith JE Richardson J Hoffman C Pilkington K. Mindfulness-based stress reduction as supportive therapy in cancer care: systematic review. J Adv Nurs. (2005) 52(3):315–27. 10.1111/j.1365-2648.2005.03592.x 16194185

[71] Surawy C Roberts J Silver A. The effect of mindfulness training on mood and measures of fatigue, activity, and quality of life in patients with chronic fatigue syndrome on a hospital waiting list: a series of exploratory studies. Behav Cogn Psychother. (2005) 33(1):103–9. 10.1017/S135246580400181X

[72] Tacón AM McComb J Caldera Y Randolph P. Mindfulness meditation, anxiety reduction, and heart disease: a pilot study. Fam Community Health. (2003) 26(1):25–33. 10.1097/00003727-200301000-00004 12802125

[73] Yook K Lee S-H Ryu M Kim K-H Choi TK Suh SY Usefulness of mindfulness-based cognitive therapy for treating insomnia in patients with anxiety disorders: a pilot study. J Nerv Ment Dis. (2008) 196(6):501. 10.1097/NMD.0b013e31817762ac 18552629

[74] Creswell JD. Mindfulness interventions. Annu Rev Psychol. (2017) 68:491–516. 10.1146/annurev-psych-042716-051139 27687118

[75] Grossman P Niemann L Schmidt S Walach H. Mindfulness-based stress reduction and health benefits. A meta-analysis. J Psychosom Res. (2004) 57(1):35–43. 10.1016/S0022-3999(03)00573-7 15256293

[76] Borquist-Conlon D Maynard B Esposito Brendel K Farina A. Mindfulness-based interventions for youth with anxiety: a systematic review and meta-analysis. Res Soc Work Pract. (2020).

[77] Roberts RL Hanley AW Garland EL. Mindfulness-Based interventions for perioperative pain management and opioid risk reduction following surgery: a stepped care approach. Am Surg. (2022):00031348221114019. 10.1177/00031348221114019 35802881

[78] Ahmadi A Hosseini M Gholizadeh L Shahboulaghi FM Brami BE. The effect of mindfulness-based cognitive therapy on anxiety of patients undergoing coronary artery bypass graft surgery: a pilot quasi randomsied controlled trail. Alternative Med. (n.d.) 7(5).

[79] Chavez JL Porucznik CA Gren LH Guan J Joyce E Brodke DS The impact of preoperative mindfulness-based stress reduction on postoperative outcomes in lumbar spine degenerative disease: 3-month and 12-month results of a pilot study. World Neurosurg. (2020) 139:e230–6. 10.1016/j.wneu.2020.03.186 32278820

[80] Gross CR Kreitzer MJ Russas V Treesak C Frazier PA Hertz MI. Mindfulness meditation to reduce symptoms after organ transplant: a pilot study. Adv Mind Body Med. (2004) 20(2):20–9. APA PsycInfo <2004 to 2005>. 15356953

[81] Hanley AW Gililland J Garland EL. To be mindful of the breath or pain: comparing two brief preoperative mindfulness techniques for total joint arthroplasty patients. J Consult Clin Psychol. (2021) 89(7):590–600. APA PsycInfo <2021>. 10.1037/ccp0000657 34165999 PMC8363593

[82] Hanley AW Garland EL. Self-transcendence predicts better pre- and postoperative outcomes in two randomized clinical trials of brief mindfulness-based interventions. Mindfulness (N Y). (2022) 13(6):1532–43. APA PsycInfo <2022 to April Week 4 2023>. 10.1007/s12671-022-01896-6

[83] Hardison ME Unger J Roll SC. Hand therapy patients’ psychosocial symptomology and interests in mindfulness: a cross-sectional study. Can J Occup Ther. (2022) 89(1):44–50. APA PsycInfo <2022 to April Week 4 2023>. 10.1177/00084174211060120 34783575

[84] Vandenbogaart E. The journey to heart transplant: Evaluating a brief mindfulness intervention on hospitalized heart failure patients’ stress, anxiety and resilience. *Dissertation Abstracts International: Section B: The Sciences and Engineering* (dissertation). (2022) 83(1-B), No-Specified. APA PsycInfo <2022 to April Week 4 2023>.

[85] Yi JL Porucznik CA Gren LH Guan J Joyce E Brodke DS The impact of preoperative mindfulness-based stress reduction on postoperative patient-reported pain, disability, quality of life, and prescription opioid use in lumbar spine degenerative disease: a pilot study. World Neurosurg. (2019) 121:e786–91. 10.1016/j.wneu.2018.09.223 30312812

[86] Failla C Marino F Bernardelli L Gaggioli A Doria G Chilà P Mediating mindfulness-based interventions with virtual reality in non-clinical populations: the state-of-the-art. Healthcare. (2022) 10(7):1220. 10.3390/healthcare10071220 35885747 PMC9316803

[87] Arpaia P D’Errico G De Paolis LT Moccaldi N Nuccetelli F. A narrative review of mindfulness-based interventions using virtual reality. Mindfulness (N Y). (2022) 13(3):556–71. APA PsycInfo <2021>. 10.1007/s12671-021-01783-6

[88] Gaggioli A Pioggia G Tartarisco G Baldus G Ferro M Cipresso P A system for automatic detection of momentary stress in naturalistic settings. Stud Health Technol Inform. (2012) 181:182–6. 10.3233/978-1-61499-121-2-182 22954852

[89] Navarro-Haro MV Modrego-Alarcon M Hoffman HG Lopez-Montoyo A Navarro-Gil M Montero-Marin J Evaluation of a mindfulness-based intervention with and without virtual reality dialectical behavior therapy mindfulness skills training for the treatment of generalized anxiety disorder in primary care: a pilot study. Front Psychol. (2019) 10. 10.3389/fpsyg.2019.00055 30745888 PMC6360930

[90] Chandrasiri A Collett J Fassbender E De Foe A. A virtual reality approach to mindfulness skills training. Virtual Real. (2020) 24(1):143–9. 10.1007/s10055-019-00380-2

[91] Modrego-Alarcon M Lopez-del-Hoyo Y Garcia-Campayo J Perez-Aranda A Navarro-Gil M Beltran-Ruiz M Efficacy of a mindfulness-based programme with and without virtual reality support to reduce stress in university students: a randomized controlled trial. Behav Res Ther. (2021) 142. 10.1016/j.brat.2021.103866 33957506

[92] Nararro-Haro MV Hoffman HG Garcia-Palacios A Sampaio M Alhalabi W Hall K The use of virtual reality to facilitate mindfulness skills training in dialectical behavioral therapy for borderline personality disorder: a case study. Front Psychol. (2016) 7. 10.3389/fpsyg.2016.01573 27853437 PMC5089996

[93] Seabrook E Kelly R Foley F Theiler S Thomas N Wadley G Understanding how virtual reality can support mindfulness practice: mixed methods study. J Med Internet Res. (2020) 22(3). APA PsycInfo <2020>. 10.2196/16106 32186519 PMC7113800

[94] Tong X Gromala D Choo A Amin A Shaw C. The virtual meditative walk: an immersive virtual environment for pain self-modulation through mindfulness-based stress reduction meditation. In: Shumaker R Lackey S, editors. Virtual, Augmented and Mixed Reality. Orlando: Springer International Publishing (2015). p. 388–97. 10.1007/978-3-319-21067-4_40

[95] Olbrecht VA O’Conor KT Williams SE Boehmer CO Marchant GW Glynn SM Transient reductions in postoperative pain and anxiety with the use of virtual reality in children. Pain Med. (2021) 22(11):2426–35. APA PsycInfo <2021>. 10.1093/pm/pnab209 34175959

[96] Bratman GN Hamilton JP Daily GC. The impacts of nature experience on human cognitive function and mental health. Ann N Y Acad Sci. (2012) 1249(1):118–36. 10.1111/j.1749-6632.2011.06400.x 22320203

[97] Marselle MR Irvine KN Warber SL. Examining group walks in nature and multiple aspects of well-being: a large-scale study. Ecopsychology. (2014) 6:134–47.

[98] Hartig T Mitchell R de Vries S Frumkin H. Nature and health. Annu Rev Public Health. (2014) 35(1):207–28. 10.1146/annurev-publhealth-032013-182443 24387090

[99] Park B Tsunetsugu Y Kasetani T Kagawa T Miyazaki Y. The physiological effects of Shinrin-Yoku (taking in the forest atmosphere or forest bathing): evidence from field experiments in 24 forests across Japan. Environ Health Prev Med. (2010) 15(1):18–26. 10.1007/s12199-009-0086-9 19568835 PMC2793346

[100] Ribeiro AI Triguero-Mas M Jardim Santos C Gómez-Nieto A Cole H Anguelovski I Exposure to nature and mental health outcomes during COVID-19 lockdown. A comparison between Portugal and Spain. Environ Int. (2021) 154:106664. 10.1016/j.envint.2021.106664 34082237 PMC8162907

[101] Berto R Barbiero G Barbiero P Senes G. An individual’s connection to nature can affect perceived restorativeness of natural environments. Some observations about biophilia. Behav Sci. (2018) 8(3):34. 10.3390/bs8030034 29510581 PMC5867487

[102] Bigloo F Kentel JA. Biophilia: a view of human-nature in an era of contradictions. Mediterr J Soc Behav Res. (2018) 2(2–3):23–6. 10.30935/mjosbr/8384

[103] Gaekwad JS Sal Moslehian A Roös PB Walker A. A meta-analysis of emotional evidence for the biophilia hypothesis and implications for biophilic design. Front Psychol. (2022) 13:750245. 10.3389/fpsyg.2022.750245 35693493 PMC9186521

[104] Bratman GN Anderson CB Berman MG Cochran B de Vries S Flanders J Nature and mental health: an ecosystem service perspective. Sci Adv. (2019) 5(7):eaax0903. 10.1126/sciadv.aax0903 31355340 PMC6656547

[105] Chavaly D Naachimuthu KP. Human-Nature connection and mental health: what do we know so far? Indian J Health Wellbeing. (2020) 11:01. 10.15614/IJHW.v11i01.18

[106] Keniger LE Gaston KJ Irvine KN Fuller RA. What are the benefits of interacting with nature? Int J Environ Res Public Health. (2013) 10(3):913–35. 10.3390/ijerph10030913 23466828 PMC3709294

[107] Kaplan R Kaplan S. The Experience of Nature: A Psychological Perspective. New York: Cambridge University Press (1989). p. xii, 340.

[108] Kaplan S. The restorative benefits of nature: toward an integrative framework. J Environ Psychol. (1995) 15(3):169–82. 10.1016/0272-4944(95)90001-2

[109] Berman MG Jonides J Kaplan S. The cognitive benefits of interacting with nature. Psychol Sci. (2008) 19(12):1207–12. 10.1111/j.1467-9280.2008.02225.x 19121124

[110] Kaplan S. Meditation, restoration, and the management of mental fatigue. Environ Behav. (2001) 33(4):480–506. 10.1177/00139160121973106

[111] Ulrich RS. View through a window may influence recovery from surgery. Science (N Y). (1984) 224(4647):420–1. 10.1126/science.6143402 6143402

[112] Dijkstra K Pieterse M Pruyn A. Physical environmental stimuli that turn healthcare facilities into healing environments through psychologically mediated effects: systematic review. J Adv Nurs. (2006) 56(2):166–81. 10.1111/j.1365-2648.2006.03990.x 17018065

[113] Kellert SR Heerwagen J Mador M. Biophilic Design: The Theory, Science, and Practice of Bringing Buildings to Life. New York: Wiley (2008).

[114] White KE. The role of nature in physiological recovery from stress: A critical examination of restorative environments theory. *Dissertation Abstracts International: Section B: The Sciences and Engineering* (dissertation). (2014). *74*(11-B(E)), No-Specified. APA PsycInfo <2014>.

[115] Kiecolt-Glaser JK Page GG Marucha PT MacCallum RC Glaser R. Psychological influences on surgical recovery: perspectives from psychoneuroimmunology. Am Psychol. (1998) 53(11):1209–18. 10.1037/0003-066X.53.11.1209 9830373

[116] Rubin HR. Status report—an investigation to determine whether the built environment affects patients’ medical outcomes. J Healthc Des. (1998) 10:11–3. 10539237

[117] Chalder M Montgomery A Hollinghurst S Cooke M Munro J Lattimer V Comparing care at walk-in centres and at accident and emergency departments: an exploration of patient choice, preference and satisfaction. Emerg Med J. (2007) 24(4):260–4. 10.1136/emj.2006.042499 17384379 PMC2658231

[118] White MP Wheeler BW Herbert S Alcock I Depledge MH. Coastal proximity and physical activity: is the coast an under-appreciated public health resource? Prev Med. (2014) 69:135–40. 10.1016/j.ypmed.2014.09.016 25284259

[119] Maas J. Green space, urbanity, and health: how strong is the relation? J Epidemiol Community Health. (2006) 60(7):587–92. 10.1136/jech.2005.043125 16790830 PMC2566234

[120] Browning MHEM Stern MJ Ardoin NM Heimlich JE Petty R Charles C. Investigating the sets of values that community members hold toward local nature centers. Environ Educ Res. (2017) 23(9):1291–306. 10.1080/13504622.2016.1177713

[121] Depledge MH Stone RJ Bird WJ. Can natural and virtual environments be used to promote improved human health and wellbeing? Environ Sci Technol. (2011) 45(11):4660–5. 10.1021/es103907m 21504154

[122] Smith J. Immersive virtual environment technology to supplement environmental perception, preference and behavior research: a review with applications. Int J Environ Res Public Health. (2015) 12(9):11486–505. 10.3390/ijerph120911486 26378565 PMC4586687

[123] Van Houwelingen-Snippe J Ben Allouch S Van Rompay TJL. Virtual reality representations of nature to improve well-being amongst older adults: a rapid review. J Technol Behav Sci. (2021) 6(3):464–85. 10.1007/s41347-021-00195-6 33688575 PMC7934124

[124] Anderson AP Mayer MD Fellows AM Cowan DR Hegel MT Buckey JC. Relaxation with immersive natural scenes presented using virtual reality. Aerosp Med Hum Perform. (2017) 88(6):520–6. 10.3357/AMHP.4747.2017 28539139

[125] Schutte NS Bhullar N Stilinović EJ Richardson K. The impact of virtual environments on restorativeness and affect. Ecopsychology. (2017) 9(1):1–7. 10.1089/eco.2016.0042

[126] Yu C-P Lee H-Y Luo X-Y. The effect of virtual reality forest and urban environments on physiological and psychological responses. Urban For Urban Green. (2018) 35:106–14. 10.1016/j.ufug.2018.08.013

[127] Chung K Lee D Park JY. Involuntary attention restoration during exposure to mobile-based 360° virtual nature in healthy adults with different levels of restorative experience: event-related potential study. J Med Internet Res. (2018) 20(11):e11152. 10.2196/11152 30504121 PMC6300039

[128] Gerber SM Jeitziner M-M Wyss P Chesham A Urwyler P Müri RM Visuo-acoustic stimulation that helps you to relax: a virtual reality setup for patients in the intensive care unit. Sci Rep. (2017) 7(1):13228. 10.1038/s41598-017-13153-1 29038450 PMC5643433

[129] Passmore H-A Holder MD. Noticing nature: individual and social benefits of a two-week intervention. J Posit Psychol. (2017) 12(6):537–46. 10.1080/17439760.2016.1221126

[130] Ivtzan I Chan CPL Gardner HE Prashar K. Linking religion and spirituality with psychological well-being: examining self-actualisation, meaning in life, and personal growth initiative. J Relig Health. (2013) 52(3):915–29. 10.1007/s10943-011-9540-2 21968697

[131] Birnie KA Noel M Parker JA Chambers CT Uman LS Kisely SR Systematic review and meta-analysis of distraction and hypnosis for needle-related pain and distress in children and adolescents. J Pediatr Psychol. (2014) 39(8):783–808. 10.1093/jpepsy/jsu029 24891439 PMC4138805

[132] Aminabadi N Erfanparast L Sohrabi A Oskouei S Naghili A. The impact of virtual reality distraction on pain and anxiety during dental treatment in 4-6 year-old children: a randomized controlled clinical trial. J Dent Res Dent Clin Dent Prospects. (2012) 6(4):117–24. 23277857 10.5681/joddd.2012.025 PMC3529924

[133] Auerbach RP Mortier P Bruffaerts R Alonso J Benjet C Cuijpers P WHO World mental health surveys international college student project: prevalence and distribution of mental disorders. J Abnorm Psychol. (2018) 127(7):623–38. APA PsycInfo <2021>. 10.1037/abn0000362 30211576 PMC6193834

[134] Allgulander C. Generalized anxiety disorder: what are we missing? Eur Neuropsychopharmacol. (2006) 16:101–8. APA PsycInfo <2019>. 10.1016/j.euroneuro.2006.04.002 16730165

[135] Altman DG. Practical Statistics for Medical Research. 1st ed. London: Chapman and Hall (1991). APA PsycInfo <2022 to April Week 4 2023>.

